# Supplementary material for: Enhancing the Therapeutic Potential of CCL2-Overexpressing Mesenchymal Stem Cells in Acute Stroke
Source: Int J Mol Sci. 2020 Oct 21;21(20):7795. doi: 10.3390/ijms21207795 (PMC7588958; doi:10.3390/ijms21207795)
Supplement: Supplementary file 1 [file ijms-21-07795-s001.pdf]

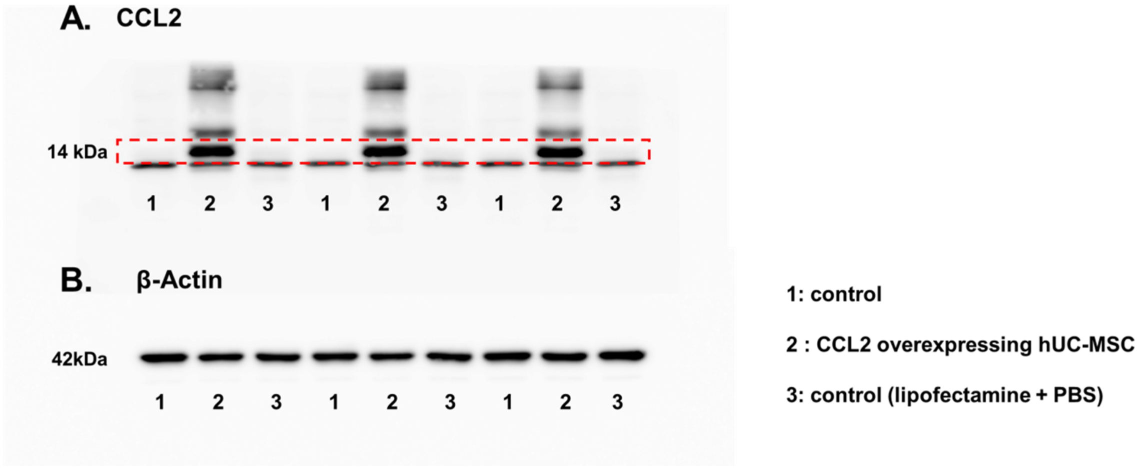

Supplementary figure 1. Uncropped membrane scans represented in Figure 1C. (A) The WB of CCL2 protein (14kDa) highlighted with red dashed box in Fig 1C. (B) Uncropped membrane scans of β-Actin. The β-Actin protein was detected by the same membrane used in the experiment for CCL2 detection. Western blot data used in Fig. 1C were used by editing control and CCL2 overexpressing hUC-MSC data.

© 2020 by the authors. Licensee MDPI, Basel, Switzerland. This article is an open access article distributed under the terms and conditions of the Creative Commons Attribution (CC BY) license (http://creativecommons.org/licenses/by/4.0/).
